# Predictors of Carbohydrate Metabolism Disorders and Lethal Outcome in Patients after Myocardial Infarction: A Place of Glucose Level

**DOI:** 10.3390/jpm13060997

**Published:** 2023-06-14

**Authors:** Yulia Kononova, Levon Abramyan, Ilia Derevitskii, Alina Babenko

**Affiliations:** World-Class Research Centre for Personalized Medicine, Almazov National Medical Research Centre, 197341 St. Petersburg, Russia

**Keywords:** myocardial infarction, stress hyperglycemia, diabetes mellitus, prediabetes, predictors of diabetes mellitus, machine learning

## Abstract

Background and aim: The aim of this study was to reveal statistical patterns in patients with acute myocardial infarction (AMI) that cause the development of carbohydrate metabolism disorders (CMD) (type 2 diabetes mellitus and prediabetes) and death within 5 years after AMI. Methods: 1079 patients who were treated with AMI in the Almazov National Medical Research Center were retrospectively selected for the study. For each patient, all data from electronic medical records were downloaded. Statistical patterns that determine the development of CMDs and death within 5 years after AMI were identified. To create and train the models used in this study, the classic methods of Data Mining, Data Exploratory Analysis, and Machine Learning were used. Results: The main predictors of mortality within 5 years after AMI were advanced age, low relative level of lymphocytes, circumflex artery lesion, and glucose level. Main predictors of CMDs were low basophils, high neutrophils, high platelet distribution width, and high blood glucose level. High values of age and glucose together were relatively independent predictors. With glucose level >11 mmol/L and age >70 years, the 5-year risk of death is about 40% and it rises with increasing glucose levels. Conclusion: The obtained results make it possible to predict the development of CMDs and death based on simple parameters that are easily available in clinical practice. Glucose level measured on the 1st day of AMI was among the most important predictors of CMDs and death.

## 1. Introduction

A history of cardiovascular disease, including acute myocardial infarction (AMI), is considered one of risk factors for type 2 diabetes mellitus (DM). Post-AMI patients often have more risk issues for type 2 DM, such as overweight, hypertension, dislipidemia, physical inactivity, advanced age, etc. [1]. Moreover, patients after myocardial infarction have an additional risk factor for DM as they need to take medications that are considered to increase the risk of diabetes (statins, thiazide diuretics, beta-blockers, etc.) [1,2].

Hyperglycemia is a common symptom in patients with AMI even without a previous history of type 2 DM. Increased glucose level occurs as a result of the stress-induced activation of neurohormonal processes [3]. Stress hyperglycemia has been shown to be a predictor of adverse outcomes in several studies [4,5]. The incidence of stress-induced hyperglycemia in patients with AMI is not well-known because the exact definition has not been established due to conflicting criteria [6,7,8]. Different studies showed 8.9–18% of patients with AMI have an admission glucose level > 11.1 mmol/L [9,10,11], while 16.8–59.5% have a glucose level > 7.8 mmol/L [9,12].

Except for transitory stress hyperglycemia, increased blood glucose level can be a sign of several glucose metabolism disorders, including newly diagnosed type 2 DM, impaired fasting glucose (IFG), and impaired glucose tolerance (IGT). Prediabetes, as well as type 2 diabetes, is associated with insulin resistance [13] and impaired insulin secretion [13,14] and leads to microvascular complications, which can have drastic implications for patients with cardiovascular conditions; survival rates from cardiovascular diseases in IGT and overt diabetes are similar [15].

Several studies have demonstrated that stress hyperglycemia is associated with an increased risk of type 2 DM—24.8% of post-AMI patients with stress hyperglycemia > 11.1 mmol/L developed type 2 DM during the 5-year period [10], and patients with hyperglycemia > 7.8 mmol/L were significantly more likely to be diagnosed with type 2 DM 6 months after AMI [14].

The aim of the present study was to uncover statistical patterns in patients with AMI that cause the development of CMDs (type 2 DM and prediabetes) and death within 5 years after AMI.

After analyzing the sustainable approaches, we decided to focus on machine learning algorithms, which provide more opportunities to achieve the results of the models, and also allow for the identification of non-linear relationships between indicators and help build better models compared to other approaches. Regarding risk assessment algorithms, Random Forest, Extreme Gradient Boosting, Support Vector Machine (SVM) Classifier, and Logistic Regression algorithms were chosen, as they are shown to be effective in estimating complication risk and event rate.

## 2. Materials and Methods

### 2.1. Patients

For this cohort retrospective study, we found 6895 patients who were treated with AMI in the Almazov National Medical Research Center from 2010 to 2021. Then, we selected 1079 patients who admitted on the first day of AMI that they had undergone surgery for myocardial infarction and were observed in the center for a 5-year period. Patients were treated with percutaneous transluminal angioplasty or coronary artery bypass grafting or mammary coronary bypass surgery. Diagnosis of AMI was made by clinical, electrocardiogram, echocardiography, and laboratory tests.

The non-inclusion criteria were as follows: diabetes or prediabetes diagnosed before or within AMI.

### 2.2. Data Collection

For patients included in the study, the diagnosis of CMD (type 2 DM, IGT, or IFG) was checked in the aforementioned 5-year period. For each selected patient, all data from electronic medical records were downloaded.

Table 1 shows the examination methods.

Laboratory parameters were measured by using standard laboratory methods. Glucose was measured in venous plasma using hexokinase method (Roche, Germany).

Statistical patterns influencing the development of CMDs and death within 5 years after AMI were identified.

### 2.3. Statistical Analysis

To create and train observation models, the classic methods of Data Mining, Data Exploratory Analysis, and Machine Learning were used. For validation, we used the following metrics: the f1 score metric, AUC-ROC, and accuracy. For the interpretation, we used the method of estimating the contribution of each predictor using the SHapley Additive exPlanations (SHAP) values. Linear algorithms were used for modelling—Logistic Regression and non-linear algorithms for Extreme Gradient Boosting (XGB), SVM, Random Forest. For each algorithm, the sample was divided into two parts: 70% of patients were used to train the models and select parameters, while 30% of the patients went undetected by the model and were used to assess the quality. The best model was chosen according to the AUC-ROC metric—the area under the ROC curve.

### 2.4. Plan of the Study

The study included nineteen steps, which can be summarized in four stages. The first stage (collecting datasets) includes:

^1.^  Unloading the data from the database;
^2.^  Unification, cleaning, and standardization of information;
^3.^  Extraction of indicators from medical texts (anamnesis, diagnoses, etc.);
^4.^  Selection of patients with developed endpoints within 5 years;
^5.^  Selection of patients who visited specialists for 5 years;
^6.^  Selection of patients without type 2 diabetes mellitus and lethal outcome over 5 years.

In the first step, data were unloaded from the center’s databases using Cache Object Scripts. The data were cleaned using regex (regular expressions). With the help of Python scripts, the information was unified and selected for each patient. Then, in the third step, the processing and extraction of the sections of medical records that contain information written in the native language was performed. Some attributes missing in the database in tabular form were extracted from these sections, such as information about the affected and stented vessels, the therapy taken, the complications of the course of the disease, and other indicators. Next, details regarding DM detection and lethal outcomes were searched for and identified. In the fifth and sixth steps, the search regarding patients who have not presented the above events and have visited specialists was made.

Next, data preprocessing took place. This step includes the following:

^1.^  Filling in data omissions;
^2.^  Value aggregation;
^3.^  Class balancing;
^4.^  Coding of categorical variables.

Data gaps were filled with medians, values were aggregated by calculating statistics, class frequencies were balanced by using oversampling method for datasets where it was necessary (diabetes), and categorical variables were encoded by one-hot-encoding and label encoding methods.

Next, the steps of the third stage took place—exploratory data analysis. This consists of the following steps:

^1.^  Statistical hypothesis testing;
^2.^  Univariate statistical analysis;
^3.^  Multivariate statistical analysis;
^4.^  Selection of statistical patterns and predictors for modeling.

Hypotheses regarding the relationship between binary attributes (complications, facts of therapy intake, gender, etc.) and target variables were tested at step eleven. The test was performed using h-square criterion. The null hypothesis (no influence of the trait on the target variable) was rejected at *p*-value > 0.05. The twelfth step was to search for variables that influence the target variables using univariate analysis with kernel density estimation (KDE) method and analysis of distribution visualizations using violin plots. Then, multivariate analysis was performed through point cloud estimation. All extracted statistical patterns, found relationships, and their direction were further used to build models and improve their quality.

The final stage was to build a model for risk assessment. This stage includes the following steps:

^1.^  Construction of initial models;
^2.^  Selection of attributes based on models;
^3.^  Selection of models;
^4.^  Interpretation;
^5.^  Validation.

In the fourteenth step, a binary classification model (and multiple models for carbohydrate metabolism disorders— type 2 DM, IGT, IFG) was built for each target variable. Further, the selection of features based on the models outlined by Shepley vector method was performed. Then, the final selection of AUC-ROC models took place. The best algorithm was validated and interpreted. The interpretation was completed by employing the SHAP, Permutation Importance, and Partial Depends Plots methods. Validation was completed using AUC-ROC metrics, accuracy (% of correct predictions), f1-measure (harmonic mean between accuracy and completeness).

## 3. Results

### 3.1. Exploratory Data Analysis

**Figure 1:**
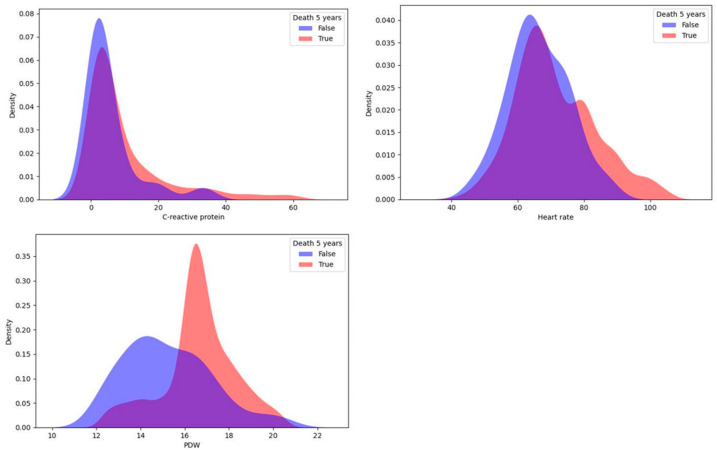
Top three most important continuous predictors of 5-year risk of death.

In this study, a univariate statistical analysis was performed using the KDE method, which proved to be good for identifying predictors among continuous variables [15]. For each target variable, the sample was divided into two parts: patients with a CMD or death within 5 years and patients without one. Then, for each continuous indicator, we reconstructed the probability density function using KDE. The differences in distributions were detected by the Kolmogorov–Smirnov two-sample test to check the null hypothesis of homogeneity in the distributions of the two samples for patients with different values of the target variable. As a result, continuous influences on the target variable were selected for each of the four target variables. The top three most important variables for 5-year risk of death identified using the KDE method are shown in Figure 1.

The KDE method revealed the following results: total cholesterol > 5 mmol/L, very low-density lipoprotein level > 1 mmol/L, and mean transvalvular gradient > 7 mm Hg increased the risk of type 2 DM development most strongly (only among continuous variables).

Important predictors of death (lethal outcome) were platelet distribution width (PDW) and C-reactive protein measured during the first day after surgery, and the average heart rate during the postinfarction period also showed a significant relationship. For each indicator, high values increased the 5-year risk of mortality.

Next, for each target variable, the association with binary variables, such as the facts of therapy intake, the presence of complications, gender, etc., was checked. The h-square criterion was used to check the null hypothesis of no relation was rejected with α < 0.05. Indicators with a confirmed effect are described in Table 2.

Next, a multivariate statistical analysis was carried out to analyze the method used to construct and generate scatter diagrams of “point clouds”. Figure 1 shows cloud points for features of age, glucose and blood lymphocytes, measurements on the first day, and measurements of the initial variable (1—lethal outcome was recorded for 5 years; 0—lethal outcome was rejected, the patient visited the center after 5-year period).

**Figure 2:**
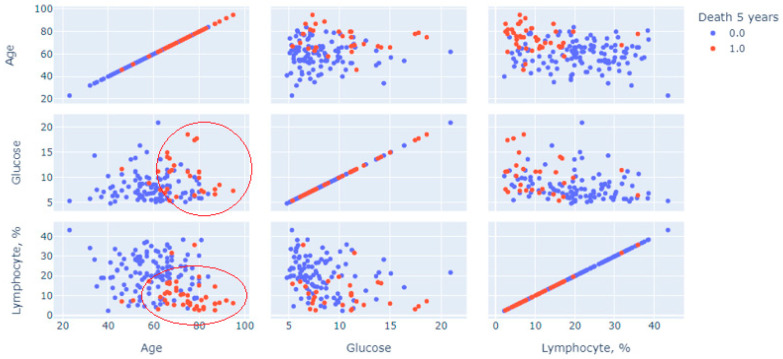
Multivariate analysis for three indicators using point cloud visualization.

The graph in Figure 2 shows a multivariate analysis of the contribution of three continuous indicators: age, glucose and relative lymphocytes level, and the prognosis of death (red dots mean death, blue dots mean absence of death). Two clusters of patients with a recorded 5-year lethal outcome are clearly distinguished on the graph. The first is in the sector limited by age above 60 years with a glucose level predominantly above 7 mmol/L; the second is age over 60 years with lymphocytes less than 20%. These pairs of restrictions were predictors of increased risk.

The identified relationships and selected predictors were used for further modelling.

### 3.2. Mortality Prediction Modelling

From the 1079 patients entered into the study, 177 patients had records in the medical information system that verified their status in the 5-year period. These patients helped inform the analysis of lethal outcome. Overall, 131 patients were alive 5 years after AMI, while 46 patients died within 5 years.

The best model was chosen according to the AUC-ROC metric—the area under the ROC curve. This metric takes values from 0 to 1. The closer the value to 1, the better the model works. An algorithm was trained, validated, and interpreted to assess the risk of death. A target value of 1 means a lethal outcome was recorded within 5 years; a target value of 0 means a lethal outcome was rejected because, after a 5-year period, the patient visited the center.

**Figure 3:**
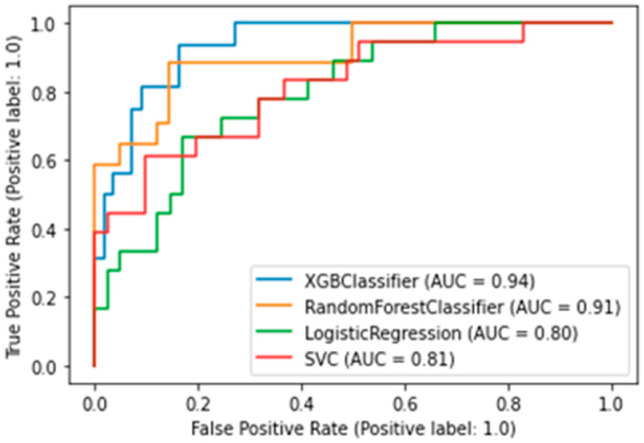
AUC-ROC plot for a binary death risk model. XGBClassifier — Extreme Gradient Boosting Classifier; SVC—Support Vector Classification).

The best algorithm was the XGBClassifier, which yielded the following metrics: AUC was 0.94 (Figure 3), accuracy (fraction of correct predictions) was 0.87, and f1-score was 0.87.

**Figure 4:**
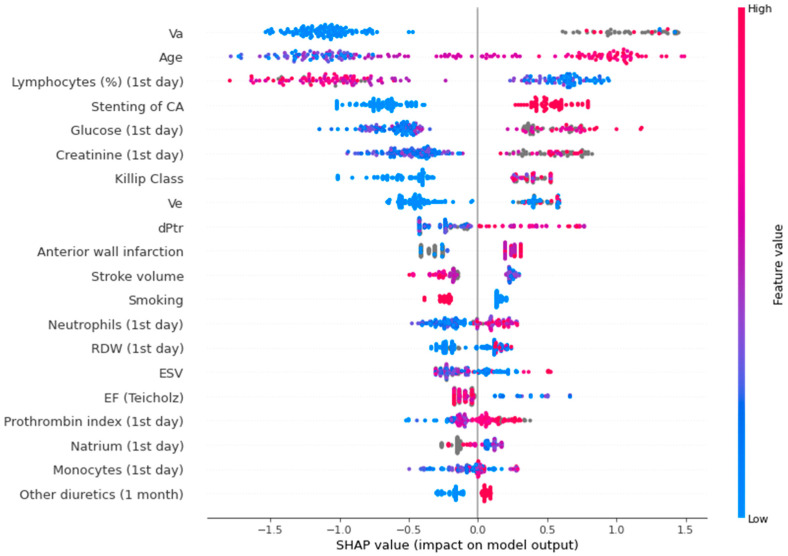
SHAP plot for the binary lethality model. Va—peak late diastolic velocity; CA—circumflex artery; Ve—peak early diastolic velocity; dPtr—transtricuspid pressure gradient; RDW—red blood cell distribution width; ESV—end systolic volume; EF—ejection fraction.

Figure 4 shows a SHAP plot for the binary lethality model.

In the SHAP plot, the value of the parameters is indicated by color. The first five indicators are the most significant: peak late diastolic velocity (association was non-linear), age (high values increase the risk, low values decrease the risk), relative level of lymphocytes (low values elevate the risk, high values decrease the risk), circumflex artery lesion (presence of this parameter increases the risk, absence - decreases the risk), glucose level (low values decrease the risk, high values increase the risk).

### 3.3. CMD Prediction Modelling

In total, 73 of the 1079 patients had newly diagnosed CMD (type 2 DM or prediabetes). The remaining 1006 patients visited an endocrinologist or cardiologist 5 years after AMI and were diagnosed with CMDs.

**Figure 5:**
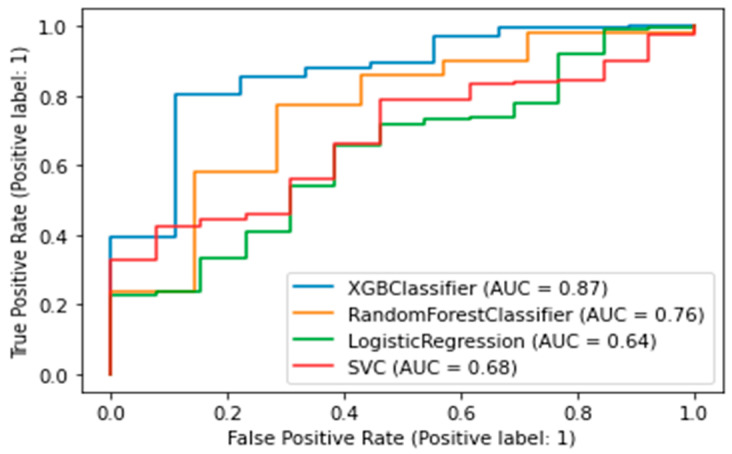
AUC-ROC plot for a binary model of diabetes. XGBClassifier—Extreme Gradient Boosting Classifier; SVC—Support Vector Classification).

A binary classifier was built for CMD. A value of target variable 1 indicates the development of diabetes or prediabetes in the five-year follow-up period; 0 indicates no CMDs was detected. The best algorithm was the XGB Classifier, which yielded the following metrics: AUC was 0.87, accuracy (fraction of correct predictions) was 0.86, and f1-score was 0.91. (Figure 5).

**Figure 6:**
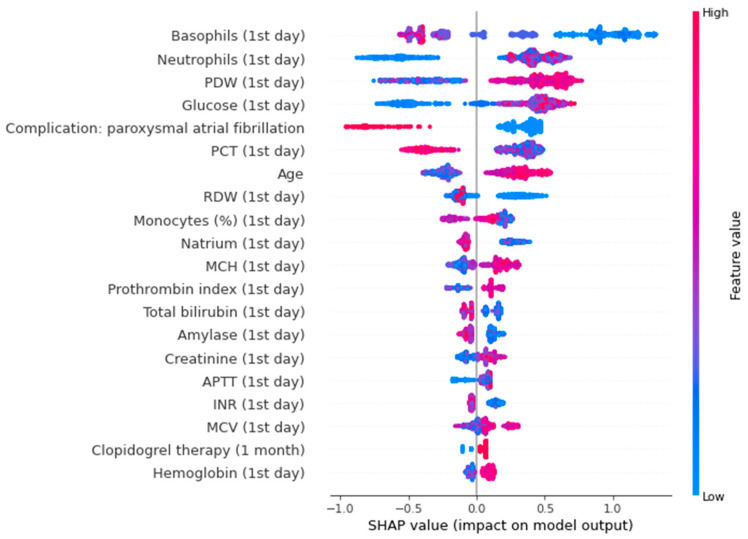
SHAP-plot for a binary model of diabetes. PDW—platelet distribution width; PCT— plateletcrit; RDW—red cell distribution width; MCH—mean cell hemoglobin; APTT—activated partial thromboplastin time; INR—international normalized ratio; MCV—mean cell volume.

The SHAP plot for the trained model, as seen in Figure 6, shows that basophils, neutrophils, PDW, and blood glucose level measured on the first day of AMI are important predictors of diabetes and prediabetes. Low basophils increase the risk, while high basophils decrease the risk. High neutrophils, PDW, blood glucose level elevate the risk of CMDs; low values of this parameters reduce the risk.

For both target variables (CVDs and death), the constructed models were characterized by a very high quality of the forecast—the accuracy rate (% of correctly predicted values of the target variable) for these models was 87 and 86%, respectively.

**Figure 7:**
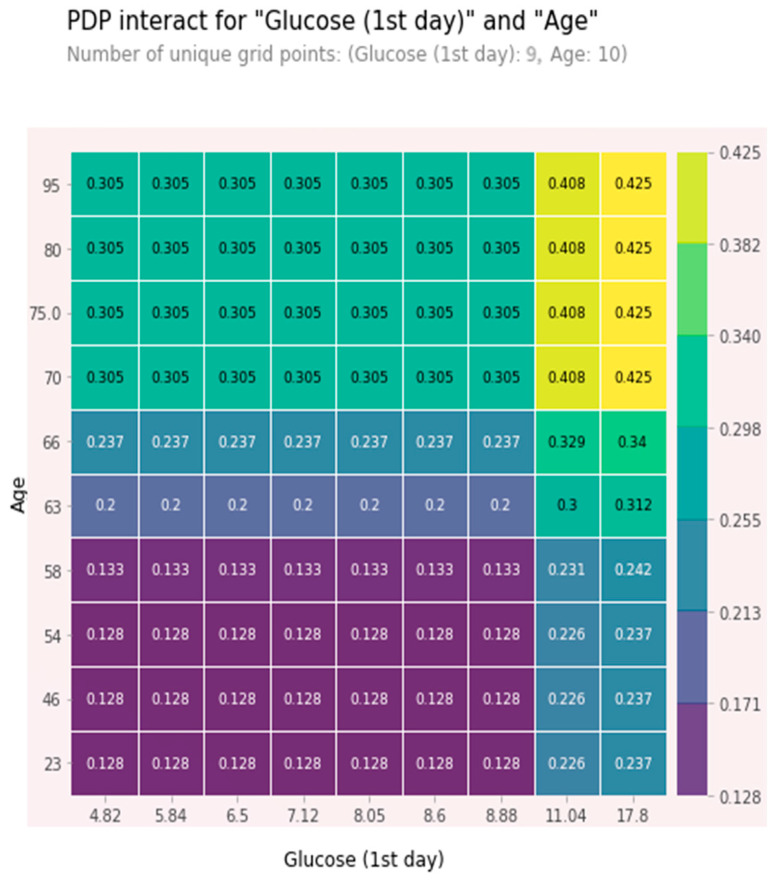
PDP plot for age and glucose.

The combined high values of age and glucose together were relatively independent predictors. These features were chosen as being among the five most significant ones, and the effect of one feature on 5-year mortality depended on the value of another feature. High values significantly increased the risk of a five-year death. At glucose value > 11 mmol/L and age > 70 years, the 5-year risk of death was about 40% and increased with glucose level growth. A partial dependence plot (PDP) for age and glucose is shown in Figure 7.

## 4. Discussion

The main findings of this study mainly include the predictors of mortality (advanced age, low relative level of lymphocytes, circumflex artery lesion, high glucose level) and predictors of CMDs (low basophils, high neutrophils, high PDW, high blood glucose level) on the 1st day of AMI.

For peak late diastolic velocity, the association was non-linear. The presence of diastolic dysfunction is diagnosed by the ratio of peak early diastolic velocity and peak late diastolic velocity. Peak late diastolic velocity in various pathological patterns of mitral blood flow can be both high and low, and from this, we can conclude that its deviations, both above and below the normal range, are associated with an increased risk of death [16].

Age has long been known as a predictor of the risk of death due to the natural increase in the frequency of complications [17]. Moreover, age rises the influence of various factors of prognosis deterioration [18].

According to the presented model, the relative level of lymphocytes on the first day of AMI is a predictor of death (high values reduce the risk, low values increase the risk). At the same time, data found in the literature describe a correlation between the level of neutrophils and the severity of coronary artery disease [19]. Neutrophilia is usually associated with relative lymphopenia, which can explain the presence of relative lymphocytes level among the predictors of the lethal outcome.

Lymphopenia has been associated with poor cardiovascular outcomes [20]. One of the mechanisms by which cardiac ischemia causes lymphopenia is the following: AMI activates the hypothalamic-pituitary-adrenal axis, leading to an increase in glucocorticoid secretion. Elevated circulating glucocorticoids induce blood lymphocyte traffic to the bone marrow via the sphingosine-1-phosphate receptor type 1, which causes lymphopenia [21]. The activation of glucocorticoids may contribute to stress hyperglycemia [5].

Circumflex artery lesion can increase the risk of death because this damage is difficult to diagnose when using an electrocardiogram [22].

The role of glucose level as a predictor of death can be explained by several reasons. Acute hyperglycemia on admission in non-diabetic patients with AMI represents previously undiagnosed abnormal glucose tolerance or reflects AMI severity.

Glucose can be a marker for earlier existing CMDs. CMDs worsen the course of AMI. The authors of [23] described various CMDs influenced the course of AMI: patients with prediabetes were characterized by multivessel coronary disease and the more frequent development of acute and chronic heart failure in AMI compared with patients without CMDs; patients with newly diagnosed DM type 2 were characterized by adverse early prognosis for myocardial infarction. Prediabetes worsens systolic functions and left ventricle diastolic parameters [24]. Another reason, explaining the role of glucose level as the predictor of death is the association of glucose level with AMI severity. The studies showed the association of glucose level with infarct size [25,26] and level of necrosis markers (creatinphosphokinase, troponin I, myoglobin) [27].

We found the most important predictors of CMDs after AMI were low basophils, high neutrophils, high PDW, high blood glucose level.

The association between glucose level at admission and the evidence of glucose metabolism disorders after AMI was shown. Results of several studies indicated this association [10,12] However, there were also results that demonstrated admission glucose level, did not predict prediabetes or diabetes development [9].

Type 2 DM is a consequence of inflammation and oxidative stress. The implementation of this relationship occurs through insulin resistance [28,29]. The platelet/lymphocyte ratio is also a strong predictor of a poorer prognosis, reflecting inflammation activity and prothrombotic status. DM is regarded as a “prothrombotic state” with increased platelet reactivity [30,31]. PDW is associated with the severity of coronary artery disease (according to the Gensini index) in patients with acute coronary syndrome [32]. We did not find any studies discussing the association between the level of basophils and development of CMDs, but the relationship between the level of basophils and glucose metabolism has been shown. Severe perioperative hyperglycemia is associated with postoperative low basophil count [33]. Moreover, as higher neutrophil counts, high PDW, and high glucose were shown to be the predictors of lethal outcome, patients with a higher level of neutrophils and PDW are more likely to have a severe course of AMI, and consequently, higher glucose level during AMI. Some studies have showed that stress hyperglycemia is associated with the development of CMDs [10,26]. In addition, patients with stress hyperglycemia during AMI are more likely to have well-known risk factors for type 2 DM (family history of type 2 DM, obesity) [34].

We did not find any studies that have evaluated the predictors of diabetes or prediabetes after AMI using machine learning methods. However, there is a study that discusses the predictors of type 2 DM development in the 5-year period in patients with cardiovascular risk using machine learning methods. In these patient cohort, parameters such as age and glucose level were among the most important features for type 2 DM prediction [35].

Limitations of our study include the absence of results pertaining to glycosylated hemoglobin during hospitalization, which could help to find and exclude all patients with preexisting AMI diabetes, and the absence of data regarding glucocorticosteroid therapy for all patients, which could influence carbohydrate metabolism.

However, some studies have demonstrated that the admission glucose level in patients with AMI does not represent previously undiagnosed abnormal glucose tolerance, and there is no significant difference in the prevalence of normal glucose tolerance among the patient groups with different admission glucose levels [9]. The difference in the results of studies on the effect of admission glucose level in patients with AMI and the development of CMDs can be explained, for example, by differences in designs, statistical methods, and the lack of a uniform definition and criteria for stress hyperglycemia. Moreover, admission glucose level does not always reflect the maximum stress elevation of glucose level because patients are not always admitted to hospital a day after an acute coronary event. This fact may influence the results of studies on the association between glucose level and the development of CMDs or adverse outcomes. In addition, not only glucose level but also other parameters may affect the results of studies aiming to identify predictors using parameters at admission. In our study, we only included patients admitted at the first day of AMI.

There are several studies [36,37] on the prediction of lethal outcome in patients with AMI using machine learning methods. A study by Oliveira et al. [36] showed that neutrophils, age, and glucose level were among the predictors of death in patients with myocardial infarction, as our results also showed. However, according to various models, the authors of this study have identified some predictors that differed from our results, such as troponin, urea, eosinophils, cardiogenic shock, and lactate dehydrogenase.

## 5. Conclusions

The level of venous serum glucose measured on the 1st day of AMI as well as the indicators of a clinical blood test were among the most significant predictors of the development of CMDs and death within 5 years after AMI. The obtained results make it possible to predict the development of CMDs and death in patients after AMI based on simple parameters available in routine clinical practice.

## Figures and Tables

**Table 1:** Examination methods.

Parameter Group	Parameters	Time of Estimation
Clinical and anamnestic	Age, sex, body mass index, smoking	Upon admission
Operation data, coronary angiography	Affected vessels, stented vessels, surgical complications	Operation data, coronary angiography
Laboratory analyses	Glucose, C-reactive protein, creatine phosphokinase, myoglobin, D-dimer, potassium, sodium, protein, creatinine, bilirubin, prothrombine time; hemoglobin, erythrocytes, hematocrit, platelets, leukocytes, neutrophils, monocytes, eosinophils, lymphocytes, basophils, red cell blood indices, platelet indices, erythrocyte sedimentation rate	First day of myocardial infarction
Lipids	total cholesterol, lipid fractions, triglycerides	4–7 days after operation
Echocardiography	Heart rate, pulmonary artery pressure, left ventricular end-diastolic volume, left ventricular end-systolic volume, ejection fraction, etc.	1 week after operation
Therapy	Renin-angiotensin-aldosteron inhibitors, statins, beta-blockers, calcium channel blockers, clopidogrel, thiazides, other diuretics, other therapy	1 month after operation

**Table 2:** Indicators identified by the h-square test influencing target variables.

Target Variable	Influencing Binary Indicator (*p*-Value)
Diabetes mellitus/prediabetes	Q wave (0.01); left anterior fascicular block (0.005); atrial fibrillation paroxysm (0.001); statin use in the post-infarction period (0.001); Killip class (0.001); thinned atrial septum (0.001); hypermobile atrial septum (0.001)
Lethal outcome	Q wave (0.001); chronic heart failure (0.001); sclerotic walls (0.02); taking the following types of therapy within 1 month after myocardial infarction—other diuretics (0.001), clopidogrel (0.003); Killip class (0.02); atrial septum (0.0001)

## Data Availability

Data can be provided upon request.
